# The Emerging Frontier of Neuromodulation for Delirium

**DOI:** 10.1016/j.neurot.2026.e01057

**Published:** 2026-09-01

**Authors:** Shixie Jiang

**Affiliations:** Department of Psychiatry, University of Florida College of Medicine, Gainesville, FL, USA

**Keywords:** Delirium, Neuromodulation, Transcranial alternating current, stimulation, Functional neuroimaging, Precision medicine

Delirium remains the most common and consequential neuropsychiatric syndrome encountered in the hospital, yet its treatment has changed remarkably little despite advances in understanding its pathophysiology. Current management appropriately emphasizes identification and treatment of precipitating medical etiologies, minimization of deliriogenic exposures, and supportive care [1]. Pharmacologic interventions may be necessary for selected symptoms or circumstances, but they generally target a limited number of neurotransmitter systems and are applied broadly across a syndrome characterized by substantial neurobiological heterogeneity [2]. Nonpharmacologic strategies similarly address salient but generalizable contributors, including immobility, sleep disruption, sensory impairment, and medication exposure, and their effectiveness depends heavily on consistent implementation within complex clinical environments [3]. Although these approaches remain important components of delirium care, neither represents a precise intervention directed at the dysfunctional neural circuitry underlying the syndrome.

This therapeutic gap is increasingly discordant with an evolving understanding of delirium neurobiology. Delirium does not appear to arise from dysfunction of a single neurotransmitter, inflammatory pathway, or anatomical region [4]. Rather, converging electrophysiologic and neuroimaging evidence implicates widespread disturbances in brain activity and functional network organization. Electroencephalographic slowing is among the most reproducible physiologic findings in delirium. Functional neuroimaging studies have further demonstrated disrupted connectivity involving prefrontal, posterior cingulate, default mode, and subcortical circuits, including fronto-thalamic and limbic systems. Collectively, these findings implicate distributed brain networks supporting attention, executive function, arousal, sensory processing, emotional regulation, and memory [[5], [6], [7], [8], [9]]. The study by van der A and colleagues in this issue of *Neurotherapeutics* builds upon this framework, reasoning that if aberrant neural oscillations are a feature of delirium, direct modulation of those oscillations may represent a fundamentally different therapeutic approach.

The authors, therefore, investigated 10-Hz transcranial alternating current stimulation (tACS), applying frontal-occipital stimulation intended to modulate alpha-frequency neural activity implicated in delirium. In this multicenter, randomized, double-blind, sham-controlled pilot study, 30 hospitalized adults with active delirium received active or sham stimulation for up to 14 days. Importantly, this was not simply an efficacy trial. Its immediate question was more fundamental: can noninvasive brain stimulation be practically and safely administered to patients while they are actively delirious? The answer is encouraging. More than 90% of participants in both groups completed their first 30-minute session, no stimulation-related serious adverse events occurred, and reported stimulation-related sensations were generally mild and transient.

That accomplishment should not be understated. Delirium is almost uniquely difficult to study prospectively [10]. The severity and manifestations of delirium can fluctuate over short periods, including disturbances in attention, arousal, cognition, perception, and psychomotor activity, while patients’ ability to participate in research may change accordingly. Patients may simultaneously require procedures, diagnostic testing, nursing interventions, or changes in medical support, and the syndrome itself may resolve before a research protocol can be completed. Indeed, these challenges are evident in the present study. Participants had a median of three eligible treatment days and completed a median of two active or three sham sessions, with adherence across eligible treatment days of approximately 70%. Recruitment was similarly demanding, with substantial attrition between screening and treatment. Thus, while tACS appears feasible to initiate during active delirium, the ability to reliably deliver repeated stimulation remains a separate and important challenge.

Nor should clinical improvement occurring during such studies be prematurely attributed to neuromodulation. Delirium is inherently dynamic, and its clinical course is influenced by numerous simultaneous changes in the underlying illness, medications, the environment, sleep, mobility, and level of care [2]. Infections are treated, sedating medications are withdrawn, metabolic abnormalities resolve, mechanical ventilation is discontinued, and patients transition through different stages of recovery. These factors complicate attribution of improvement to any experimental intervention, particularly over several days. Importantly, however, this does not imply that correction of the precipitating medical illness must remain the only means of treating delirium itself. Indeed, the promise of neuromodulation lies partly in the possibility that the dysfunctional brain circuits underlying delirium may eventually become direct therapeutic targets, even while the precipitating illness is still being treated. The investigators appropriately emphasize that their pilot was neither designed nor powered to determine whether tACS contributed to delirium resolution amid concurrent medical treatment. The next immediate step is, therefore, clear: adequately powered trials must establish whether neuromodulation produces clinically meaningful changes in delirium severity, duration, or other patient-centered outcomes beyond concurrent medical treatment.

Yet efficacy alone should not represent the endpoint of this emerging field. Delirium is defined syndromically, but this shared clinical diagnosis is unlikely to reflect a uniform underlying neurobiology [4]. Two patients meeting the same diagnostic criteria may differ substantially in level of arousal, psychomotor phenotype, attentional impairment, cognitive profile, precipitating etiology, premorbid vulnerability, medication exposure, and illness severity. These clinical differences may be accompanied by equally meaningful differences in the neural circuitry disrupted during delirium. A standardized stimulation protocol applied to every patient necessarily assumes a degree of biological homogeneity that may ultimately prove unrealistic.

This is where neuromodulation may become particularly transformative. Its promise is not simply that it offers another treatment to add to the existing delirium armamentarium. Rather, neuromodulation creates the possibility of directly targeting dysfunctional neural circuits and, eventually, selecting those targets according to the biology of the individual patient. Achieving that goal will first require better characterization of the brain during delirium: identifying reproducible patterns of network dysfunction, defining their relationships with clinically meaningful phenotypes and etiologies, and determining which circuit disturbances contribute to the clinical manifestations of delirium and, therefore, represent meaningful therapeutic targets.

tACS represents only one potential approach to neuromodulation in delirium. Other noninvasive modalities, including transcranial direct current stimulation (tDCS) and transcranial magnetic stimulation (TMS), differ in their spatial characteristics, mechanisms of action, and approaches to stimulation delivery and, therefore, warrant investigation in delirium. It remains too early to determine whether particular modalities, stimulation parameters, or targets will prove more effective for delirium or for specific patient subgroups. Advances in electrophysiology, functional neuroimaging, computational modeling, and image-guided neuromodulation may eventually allow stimulation targets to be informed by patterns of circuit dysfunction identified during delirium. Future mechanistic studies should, therefore, ask not only whether patients improve following stimulation, but whether the targeted circuit was successfully engaged and whether such engagement relates to clinical response.

By demonstrating that rigorous, sham-controlled neuromodulation research can be conducted during active delirium, van der A and colleagues have opened the door to these next questions. For decades, delirium treatment has largely approached the brain indirectly—through correction of precipitating medical insults, minimization of iatrogenic contributors, supportive care, and management of the syndrome’s clinical manifestations. The possibility of directly interrogating and therapeutically modifying the circuits underlying delirium represents a different paradigm. The work ahead will require rigorous efficacy trials alongside increasingly sophisticated investigation of delirium neurobiology, heterogeneity, and circuit-level target engagement. As tools for mapping and modulating human brain function increasingly reach the bedside, a syndrome once defined almost entirely by clinical observation may increasingly become one whose underlying circuitry can be measured, targeted, and ultimately treated with greater precision.

## Author contributions

Dr. Jiang was responsible all activities involved in this commentary, including conception, writing, editing, organization, and submission of the final product.

## Declaration of interests

The authors declare the following financial interests/personal relationships which may be considered as potential competing interests:

Dr. Jiang serves as an Associate Editor of General Hospital Psychiatry (Elsevier) and receives an honorarium for his work as an Editor. If there are other authors, they declare that they have no known competing financial interests or personal relationships that could have appeared to influence the work reported in this paper.
